# The bidirectional relationship between growth and appetite regulation in the first year of life

**DOI:** 10.1093/eurpub/ckac129.696

**Published:** 2022-10-25

**Authors:** C Demetriou, A Singhal, J Lanigan, A Mahmood, C Vichas, D Hileti

**Affiliations:** 1 Department of Primary Care and Population Health, University of Nicosia Medical School, Nicosia, Cyprus; 2 Childhood Nutrition Research Centre, UCL GOS Institute of Child Health, London, UK; 3 Department of Life Sciences, University of Nicosia, Nicosia, Cyprus

## Abstract

Childhood obesity is a public health crisis. Even though appetite traits in infancy were associated with childhood adiposity, whether early weight gain can influence later appetite has not been researched. Our aim was to prospectively examine the bidirectional association between growth and appetite traits during the first year of life. We followed up 450 healthy term infants for 12 months (m). Appetite traits at 4 weeks (wk), 6m and 12m were assessed using the Baby and Child Eating Behaviour Questionnaires. Infant feeding, anthropometric, socioeconomic and demographic data were also collected. Infant weight-for-age z-scores (WFAZ) were calculated using the WHO 2006 growth reference. Growth was assessed as conditional WFAZ change (cWFAZc) by saving the residuals from linear regression models of WFAZ at each successive time point versus WFAZ at the earlier time point. Multivariable linear regression was used to analyse bidirectional associations between cWFAZc (0-4wk, 4wk-6m, 6-12m) and appetite traits Enjoyment of Food (EF), Food Responsiveness (FR), Satiety Responsiveness (SR) and Slowness in Eating (SE) at 4wk, 6m and 12m. All models were adjusted for relevant confounders. At 4wk, SR score was associated with lower (β:-0.16; 95% CI:-0.28,-0.03), and FR score with higher (β:0.10; 95% CI:0.01,0.19) cWFAZc from 4wk to 12m. SR score at 6m was inversely associated with cWFAZc from 6-12m (β:-0.09; 95% CI:-0.16,-0.01). Conversely, higher cWFAZc between 4wk-6m was associated with higher EF (β:0.10; 95% CI:0.01,0.19) and FR (β:0.16; 95% CI:0.04,0.29) scores at 12m. cWFAZc between 6m-12m was inversely associated with SR at 12m (β:-0.18; 95% CI:-0.35,-0.01). Our results suggest that the growth acceleration hypothesis, where faster growth in infancy leads to later obesity, may be mediated by an up-regulation of appetite traits at 12m. This highlights the public health importance of avoiding growth acceleration in infancy as a way to curb the childhood obesity epidemic.

**Key messages:**

• Weight gain in early infancy impacts appetite regulation in the first year of life and up-regulation of appetite traits at 12 months predisposes to childhood obesity.

• Avoiding growth acceleration in infancy can decrease the risk for childhood obesity.

